# Trends in Antiretroviral Regimen Complexity Among Medicare Beneficiaries With HIV, 2014-2018

**DOI:** 10.1093/geroni/igab046.668

**Published:** 2021-12-17

**Authors:** Sean Fleming, Linda Wastila

**Affiliations:** 1 University of Maryland Baltimore, University of Maryland Baltimore, Maryland, United States; 2 University of Maryland School of Pharmacy, Baltimore, Maryland, United States

## Abstract

Little is known about antiretroviral therapy (ART) patterns among Medicare beneficiaries with Human Immunodeficiency Virus (HIV). ART has significant implications for spending in Medicare Part D as use of single-tablet regimens (STR) grows, generic availability remains low, and price increases for branded therapies consistently exceed inflation. The objective of this study is to detail patterns of STR utilization among Medicare beneficiaries with HIV. We conducted a retrospective trend analysis using a 5% sample of Medicare Chronic Conditions Data Warehouse, 2014-2018. We included each person-month that fee-for-service beneficiaries with HIV had Parts A, B, and D coverage. Trends in annual prevalence of STR overall, by ART class, and by age, sex, and race subgroups were estimated. The study included 9,509 beneficiaries who contributed 345,708 person-months to the analysis. The prevalence of STR increased from 21.8% (95%CI, 21.5-22.1) in 2014 to 44.6% (95%CI, 44.3-45.0) in 2018 (p <0.0001), an increase of 104.6%. Integrase strand transfer inhibitors (INSTI) saw the largest increase in utilization between 2014 (4.4% [95%CI 4.2-4.5]) and 2018 (35.1% [95%CI 34.8-35.4]) (p<0.0001), a 701.8% increase. All sociodemographic subgroups experienced similar growth in STR use between 2014 and 2018. STR and INSTI utilization increased significantly over the study period, suggesting increased ART spending under Part D. Although increasing availability of generic multi-tablet ART regimens (MTR) may offer cost-savings, further research is needed comparing generic MTR to branded STR with regards to patient preferences, adherence, healthcare resource utilization, and total costs in the growing population of Medicare beneficiaries with HIV.

